# Overexpression of Semaphorin-3A and Semaphorin-4D in the Peripheral Blood from Newly Diagnosed Patients with Chronic Lymphocytic Leukemia

**Published:** 2019-01-01

**Authors:** Somayeh Parsa, Sedigheh Sharifzadeh, Ahmad Monabati, Noorossadat Seyyedi, Reza Ranjbaran, Mohammad Reza Baghbani, Maryam Nemati, Abdollah Jafarzadeh

**Affiliations:** 1 Department of Immunology, School of Medicine, Rafsanjan University of Medical Sciences, Rafsanjan, Iran; 2 Diagnostic Laboratory Science and Technology Research Center, School of Paramedical Sciences, Shiraz University of Medical Sciences, Shiraz, Iran; 3 Hematology Research Center, Shiraz University of Medical Sciences, Shiraz, Iran; 4 Department of Laboratory Sciences, School of Para-Medicine, Kerman University of Medical Sciences, Kerman, Iran; 5 Molecular Medicine Research Center, Research Institute of Basic Medical Sciences, Rafsanjan University of Medical Sciences, Rafsanjan, Iran; 6 Department of Immunology, School of Medicine, Kerman University of Medical Sciences, Kerman, Iran

**Keywords:** Chronic lymphocytic leukemia, Semaphorins, SEMA3A, SEMA4A, SEMA4D

## Abstract

**Background:** Semaphorins play prominent roles in physiological and pathological processes such as vascular development, tumor growth and immune responses. Semaphorins have different roles in various kinds of cancers, but there is no study concerning their expression in the chronic lymphocytic leukemia (CLL). This study aimed to assess the SEMA3A, SEMA4A and SEMA4D expression in patients with CLL.

**Materials and Methods: **Peripheral blood specimens were collected from 30 newly-diagnosed untreated patients with CLL and 30 healthy subjects as a control group. The SEMA3A, SEMA4A and SEMA4D expression was determined by real-time PCR method.

**Results: **The fold change expression of SEMA3A and SEMA4D was 7.58 ± 2.66 and 3.20 ± 0.99 in patients with CLL, and was 1.01 ± 0.31 and 1.00 ± 0.27 in healthy subjects, respectively. The CLL patients expressed higher amounts of SEMA3A and SEMA4D in comparison with healthy subjects (P<0.02 and P<0.03, respectively). The fold change expression of SEMA3A in patients with stage II (11.12 ± 5.35) was also higher than patients with stage I (4.49 ± 1.61, P<0.05). No significant difference was also observed in the expression of SEMA4A and SEMA4D between patients with stage I and stage II CLL. In both CLL and control groups, the fold change expression of SEMA3A was higher in men than in women (P<0.03 and P<0.02, respectively).

**Conclusion: **The results of the study indicated elevated expression of the SEMA3A and SEMA4D in patients with CLL. The SEMA3A expression was influenced by tumor stage and gender of participants.

## Introduction

 Chronic lymphocytic leukemia (CLL) is the most frequent type of leukemia in adults with higher occurrence in men than women (1.7:1)^1^. CLL is characterized by the expansion of monoclonal of B lymphocytes expressing B-cell markers such as CD19, low amounts of CD20, and CD23^2^. CLL cells also exhibit unusual expression of CD5 (a T-cell marker), but they don’t express CD10^2^. Recently, CD200 has been considered as a prominent marker for differential diagnosis of CLL from other lymphoproliferative malignancies^2^.

The genetic parameters, occupational and lifestyle factors as well as infectious agents, especially hepatitis C virus, are related with CLL development^2^^,^^3^. In CLL, the activation of memory/marginal zone B-cells by chronic antigen stimulation and genetic damages results in the monoclonal B cell lymphocytosis. The happening of the more genetic aberrations leads to the oncogenic transformation of B cells, which is considered as frank CLL^4^. 

Semaphorin is originated from the semaphore meaning the convey information through a signaling system^5^. The main characteristic of the semaphorins is the presence of a sema domain, composed of approximately 500 amino acids, placed close to their N-terminus^6^. The sema domain plays a principle role in semaphorin activity as it determines the specificity of the receptor binding^7^. The semaphorin family is gathered into eight subclasses, of which subclasses 1 and 2 possess invertebrate semaphorins, whereas subclasses 3-7 possess the vertebrate semaphorins. The eighth subclass of semaphorins possesses viral semaphorins^6^. In normal physiological situations, semaphorins are produced as secretary or membrane- linked proteins^6^^,^^7^. Semaphorins play principal roles in the developmental process during fetal period^8^. The expression of semaphorins is reduced after maturation, but their expression changes in pathologic conditions^9^^,^^10^. Plexins and neuropilins serve as the major receptors for most semaphorins^6^. 

Several semaphorins, including SEMA3A, SEMA4A and SEMA4D named immune semaphorins are mainly involved in the immune responses by influencing on the immune cell-cell interactions or migration, including T-cells and dendritic cells (DCs) ^11^.

SEMA3A binds to neuropilin-1 and acts as a strong suppressor of angiogenesis and tumor progression in various kinds of solid tumors^11^. Down-regulation of SEMA3A expression in cancerous cells enhances angiogenesis and tumor progression, representing that it acts as an endogenous suppressor of the angiogenesis^6^.  

SEMA4A is a membrane-linked semaphorin that binds to the plexin-D1 and T-cell immunoglobulin domain and mucin domain (TIM)-2 expressed on activated T cells^11^. SEMA4A enhances the angiogenesis by enhancing VEGF expression.^11^ SEMA4A also increases the macrophage migration and induces the VEGF expression, thereby promoting angiogenesis^6^^,^^12^. Moreover, SEMA4A binds to neuropilin-1 on T-regulatory (Treg) cells and enhances their survival^12^.

SEMA4D is another membrane-related semaphorin that binds to plexin-B1, plexin-B2 and CD72 receptors^6^. SEMA4D has also immunoregulatory activities and enhances proliferative response and cytokine secretion by CD4^+^ T-cells, induces DC maturation and up-regulates the expression MHC class II and co-stimulatory molecules CD80 and CD86 on DCs and enhances natural killer (NK) cell activity^11^. In the tumor microenvironment, SEMA4D is largely synthesized by tumor associated macrophages^11^. Although SEMA4D activates anti-tumor immune responses that may repress tumor growth, it also potentiates angiogenesis and tumor growth^11^. Semaphorins have opposite functions in different kinds of cancers and their activities in tumor development are exerted through a tumor type and dose dependent manner^13^. Semaphorins are synthesized by the cancerous cells and by leukocytes accumulated in the tumor microenvironment such as macrophages, T cells and DCs^6^^,^^7^^,^^11^. It should be noted that many types of immune abnormalities occur in patients with malignant disease^14^^-^^18^. Immune-related semaphorins such as SEMA3A, SEMA4A and SEMA4D may also affect tumor progression via the influencing the immune system. No study was found concerning the expression of semaphorins in CLL. Therefore, the aim of this study was to evaluate the expression of the aforementioned immune-related semaphorins in blood samples obtained from the CLL patients to clarify a possible association. 

## MATERIALS AND METHODS

**Subjects**

A total of 30 untreated CLL patients (22 men and 8 women) with the age range of 42-80 years (mean age: 62.20 ± 11.46 years) attending the Hematology and Oncology Unit of Shahid Faghihi Hospital, affiliated to Shiraz University of Medical Sciences were enrolled in this study. All study participants were newly-diagnosed cases and had not already received any treatment. CLL diagnosis was made by an expert hematopathologist according to the criteria such as lymphocytosis of 5× 10^9^/L in peripheral blood, cell morphology and immunophenotyping using CD5, CD19, CD20 and CD23 markers which were in agreement with World Health Organization (WHO) guidelines^19^. The CLL staging was carried out according to the Rai staging guideline and National Cancer Institute Working Group criteria^20^. Accordingly, 16 CLL patients were in stage I, and 14 CLL patients were in stage II. Moreover, 30 healthy age- and sex-matched individuals (20 men and 10 women) with the age range of 42-76 years (60.90 ± 8.99 years) were recruited. The healthy individuals were in good health without medication, current disease and history of hematologic malignancies or other hematologic abnormalities. A peripheral blood specimens were taken from each participant after obtaining written informed consents. The protocol was also approved by the Ethics Committee of Rafsanjan University of Medical Sciences (Ethical code number: IR.RUMS.REC.1394.168). 

As there was no previous study concerning the assessment of semaphorins in patients with CLL, therefore, the sample size was determined according to data of SEMA3A expression obtained from our pilot and another study^21^. The mean ± SD was 1.00 ± 0.50 in the control group and 3.00 ± 2.50 in patients. The type I error (α), 1-α, and the study power were considered as 0.05, 0.95 and 80.0%, respectively.

**RNA extraction**

Total RNA extraction was performed from peripheral blood samples using RNX-plus solution (Cinnagen, Tehran, Iran) according to the manufacturer's protocol. Briefly, 1 ml of cold RNX-Plus reagent (Guanidine/phenol solution) was added to a 2-ml tube containing 200 µl blood sample and incubated at room temperature for 5 minutes. Afterwards, 200 μl of Chloroform was added, mixed for 15 seconds by shaking and incubated on ice for 5 minutes. After centrifugation (at 12000 rpm, at 4 ºC for 15 minutes), the aqueous phase was transferred to a new RNase-free 1.5 ml tube, then the equal volume of isopropanol was added and incubated on ice for 15 minutes. The mixture was centrifuged (at 12000 rpm at 4 ºC for 15 minutes), the supernatant was discarded, and 1 ml of 75% ethanol was added to the pellet and centrifuged again (at 4 ºC for 8 minutes, at 7500 rpm). Finally, the supernatant was discarded and the pellet dissolved in 50 μl of diethyl pyrocarbonate (DEPC)-treated water. The electrophoresis on an ethidium bromide pre-treated agarose gel was used for estimation of the quality of extracted RNA. The RNA purity was assessed by measuring absorption and calculation of 260/280 ratio using a spectrophotometer. 

**cDNA synthesis**

The complementary DNA (cDNA) was synthesized from the extracted RNA template using a cDNA synthesis kit (Bioneer, Daejeon, Korea) containing both random hexamer and oligo (dT) and primers. Briefly, 2 μl of oligo (dT) primer, 2 μl of 2.5 mM stock of dNTPs and 16 μl of the extracted RNA in DEPC-treated water were mixed and incubated at 70 °C for 5 minutes and then on ice for 3 minutes. The reverse transcription process was done based on the following protocol: 70°C for 10 min, 20°C for 1 minute (cooling stage), addition of reverse transcriptase enzyme, 42°C for 60 minutes, and finally the protocol was completed by a step at 95°C for 10 minutes to halt the reverse transcriptase activity. 

**Quantitative real-time PCR**

The analysis of the gene expression of SEMA3A, SEMA4A and SEMA4D was performed using a real-time PCR system (Applied Biosystems, CA, USA). Primers were synthesized by the Bioneer Company (Bioneer, Daejeon, Korea) (Table 1). Briefly, 12.5 µl of SYBR Green Master Mix (Applied Biosystems, CA, USA), 200 ng of template cDNA (in 1 µl), 2 µl of suitable primers (10 pmol stock) and 9.5 µl of DEPC-treated water were loaded per well on a real-time PCR optical plate. The plate was covered with an adhesive film and placed in the real-time PCR system. The thermal cycling program was entailed: an initial heating at 95°C for 10 min, 50 cycles of 95°C for 15 s and 56°C for 20 s and eventually 60°C for 1 minute. The -actin gene was applied as a housekeeping gene for normalization of the amplified signal of the target genes. Gene expressions of SEMA3A, SEMA4A and SEMA4D were determined using 2^-∆∆CT^ method and expressed as units relative to the amount of β-actin mRNA. 

**Table 1:** The used primer for assessment of the mRNA expression of semaphorins

**Genes**	**Primer sequences**	**Product ** **size ** **(bp)**
SEMA3A	Forward primer: 5- CAGAAGATGGACAGTATGATGTT-3 Reverse primer: 5-GTTGTTGCTGCTTAGTGGAA-3	180
SEMA4A	Forward primer: 5-GCTTGTACCTTCATTGAACTTC- 3 Reverse primer: 5- CATAGTACCAGAATAGAGCATCC-3	147
SEMA4D	Forward primer: 5-GAAGCAGCATGAGGTGTATT-3 Reverse primer: 5-GGATGTTAAGTTCAGGTGGTC-3	187
Beta- actin	Forward primer: 5-ATCGTGCGTGACATTAAGGAG- 3 Reverse primer: 5-GAAGGAAGGCTGGAAGAGTG- 3	177

**Statistical analysis**

Results are presented as means ± SEM. Statistical comparison was determined by using ANOVA and Student's t-test. The P values of less than 0.05 were considered significant. The data were analyzed using the SPSS software, version 22 (SPSS Inc, Chicago, IL, USA). 

## Results

 The differences in gender ratio and age between CLL patients and healthy individuals were not significant (P=0.57 and P=0.75, respectively). 

**Expression of SEMA3A in CLL patients and control group**

The fold change expression of SEMA3A in patients with CLL (7.58 ± 2.66) was significantly higher than in in healthy subjects (1.01 ± 0.31; P<0.02) (Table 2). 

**Table 2:** Comparison of the mRNA expression of semaphorins in peripheral blood samples from healthy individuals and CLL patients.

Semaphorins	CLL patients			Healthy control group	
	Stage I	Stage II	Total		P value
SEMA3A expression†	4.49 ± 1.61	11.12 ± 5.35	7.58 ± 2.66	1.01 ± 0.31	*= 0.05 **=0.01 ***=0.001
SEMA4A expression†	1.87 ± 0.72	1.26 ± 0.47	1.59 ± 0.44	1.00 ± 0.14	*=0.48 **=0.25 ***=0.61
SEMA4D expression†	2.96 ± 0.72	3.48 ± 2.02	3.20 ± 0.99	1.00 ± 0.27	*=0.49 **=0.01 ***=0.01

The *, ** and *** represent the difference of the fold change expression of a specified semaphorin between stage I and stage II patients, between stage I patients and control group, and between stage II patients and control group, respectively.

† The amounts of the semaphprin expression were assessed as fold change and expressed as mean ± SEM.

**Figure 1:**
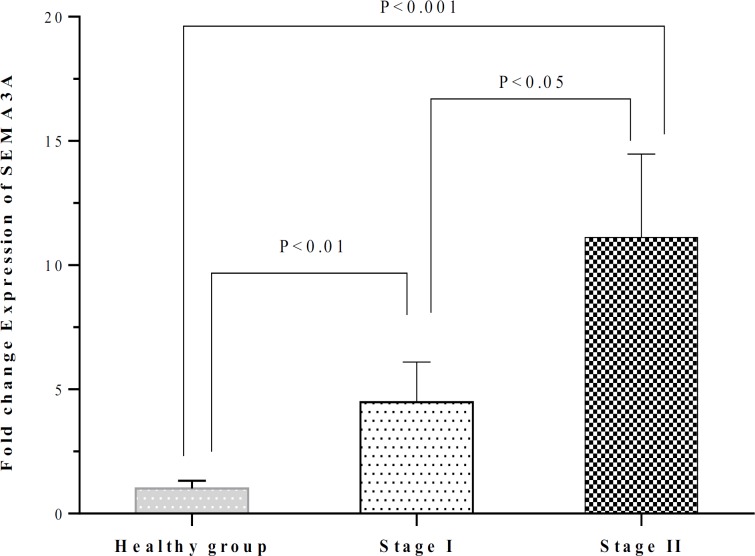
Comparison of the SEMA3A expression between healthy individuals and CLL patients who were in stages I and II. The SEMA3A expression in patients with stage I and stage II was higher than healthy subjects (P<0.01 and P<0.001, respectively). The SEMA3A expression in patients with stage II was also higher those with stage I (P<0.05).

The fold change expression of SEMA3A was 4.49 ± 1.61 in CLL patients who were in stage I and 11.12 ± 5.35 in CLL patients who were in stage II. The SEMA3A expression in both subgroups of patients with stage I and stage II was significantly higher than healthy subjects (P<0.01 and P<0.001, respectively). The SEMA3A expression in stage II patients was also higher than in stage I patients (P<0.05) (Table 2 and Figure 1). 

**Figure 2:**
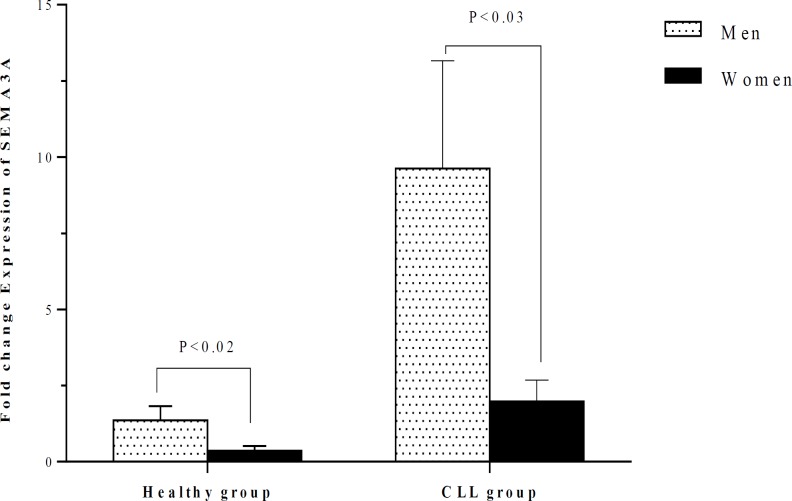
Comparison of the SEMA3A expression between healthy individuals and CLL patients according to their gender. In both CLL and control groups, the expression of SEMA3A in men was higher than women (P<0.03 and P<0.02, respectively).

In both CLL and control groups, the fold change expression of SEMA3A in men was higher than in women (P<0.03 and P<0.02, respectively) (Figure 2). The expression of SEMA3A in men and women with CLL was significantly higher than in healthy individuals of the same gender (P<0.001 and P<0.01, respectively).

**Expression of SEMA4A in CLL patients and control group**

**Figure 3:**
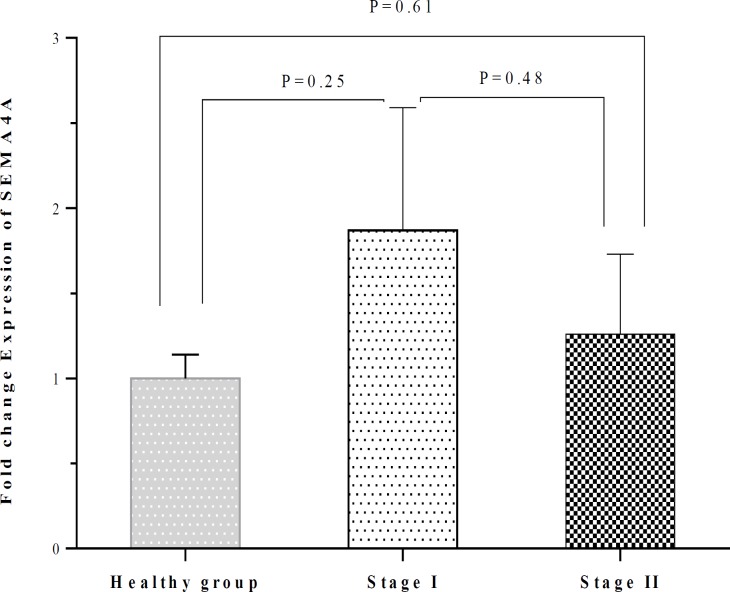
Comparison of the SEMA4A expression between healthy individuals and CLL patients who were in stages I and II. No significant difference was also observed between patients with stage I and stage II regarding the SEMA4A expression SEMA4A. The expression of SEMA4A in patients with stage I and stage II was not significantly differed as compared with healthy subjects.

**Figure 4:**
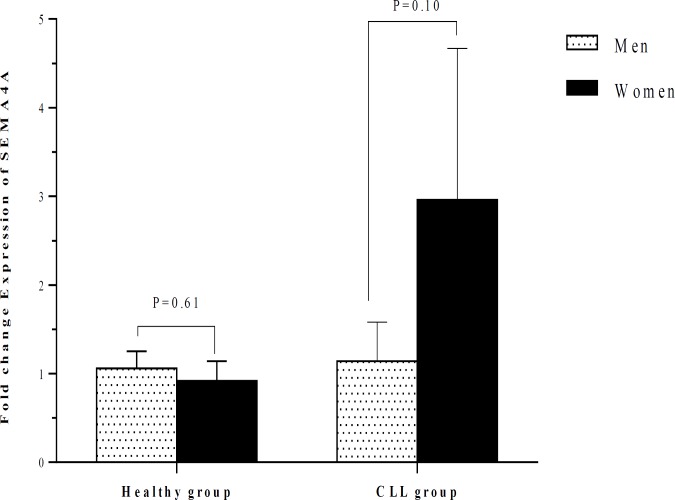
Comparison of the SEMA4A expression between healthy individuals and CLL patients according to their gender. The expression of SEMA4A did not significantly differ between men and women neither in CLL patients nor in the healthy group.

There was no significant difference between patients with CLL and healthy subjects concerning the SEMA4A expression (1.59 ± 0.54 vs 1.00 ± 0.14; P=0.25) (Table 2). No significant difference was also observed between patients with stage I and stage II regarding the expression of SEMA4A, although the amounts of this semaphorin were higher in patients with stage I. The SEMA4A expression in patients with stage I and stage II was not significantly different compared to healthy subjects (Table 2 and Figure 3). The SEMA4A expression did not significantly differ between men and women neither in CLL patients nor in the healthy group (Table 3 and Figure 4). The SEMA4A expression did not significantly differ between male patients with CLL and control group patients. The expression of SEMA4A in women with CLL was higher than in healthy women, but the difference was not statistically significant (P=0.10). 

**Expression of SEMA4D in CLL patients and control group**

**Figure 5:**
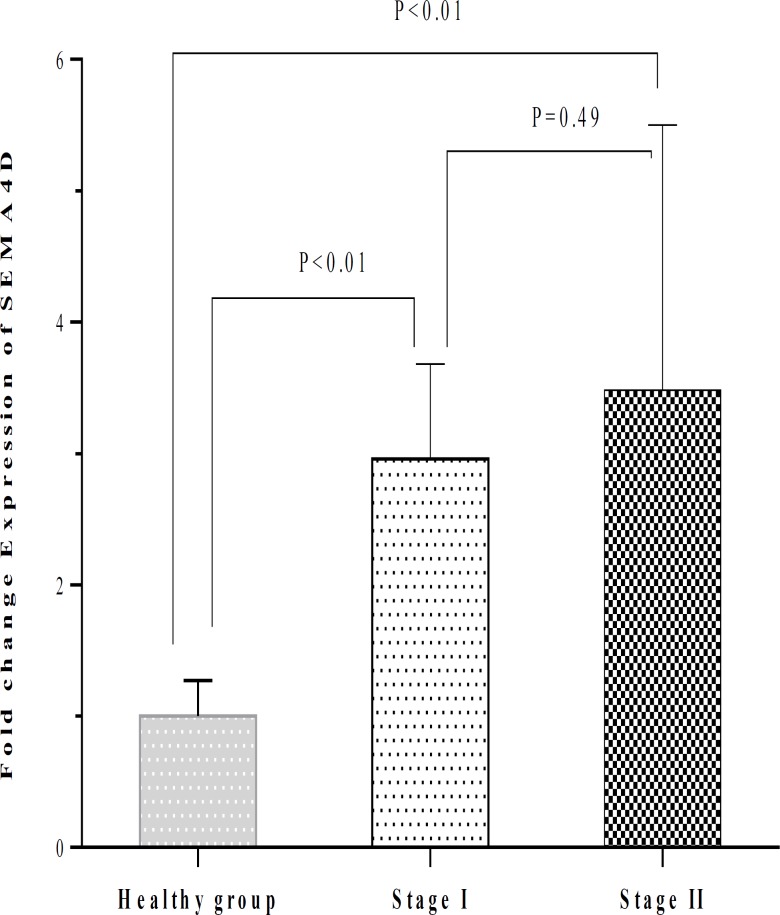
Comparison of the SEMA4D expression between healthy individuals and CLL patients who were in stages I and II. The SEMA4D expression in CLL patients with stage I and stage II was higher than control group (P<0.01). No significant difference was found between patients with stage I and stage II regarding the SEMA4D expression.

**Figure 6:**
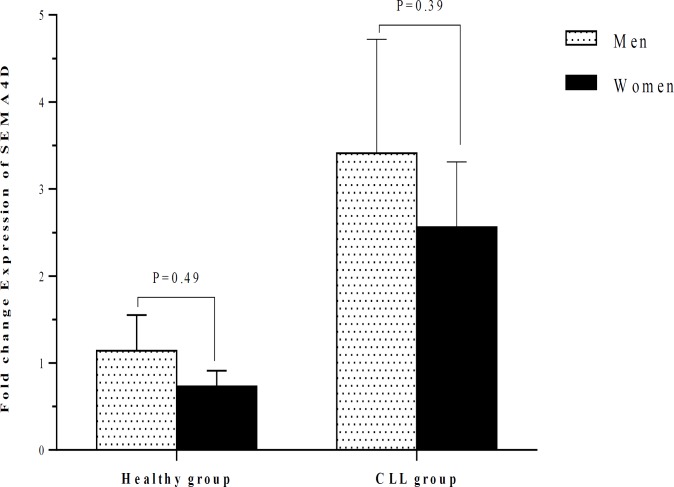
Comparison of the SEMA4D expression between healthy individuals and CLL patients according to their gender. The expression of SEMA4D did not significantly differ between men and women neither in CLL patients nor in the healthy group.

The fold change expression of SEMA4D in CLL patients (3.20 ± 0.99) was significantly higher in comparison with control group (1.00 ± 0.27; P<0.03) (Table 2). The fold change expression of SEMA4D was 2.96 ± 0.72 in patients who were in stage I and 3.48 ± 2.02 in patients who were in stage II. The SEMA4D expression in CLL patients with stage I and stage II was significantly higher than in healthy group (both with P<0.01). No significant difference was observed between patients with stage I and stage II regarding the SEMA4D expression (Table 2 and Figure 5). The expression of SEMA4D did not also change between men and women neither in CLL patients nor in the healthy individuals (Table 3 and Figure 6). The SEMA4D expression in men and women with CLL was significantly higher than healthy individuals of the same gender (P<0.01 and P<0.01, respectively).

**Table 3:** Comparison of the mRNA expression of semaphorins in peripheral blood samples from healthy individuals and CLL patients according to gender.

**Groups**	**Gender**	**Number**	**SEMA3A ** **expression** †	**SEMA4A expression** †	**SEMA4D ** **expression** †	**P value**
CLL patients	Men	22	9.62 ± 3.54	1.14 ± 0.44	3.41 ± 1.31	*=0.03 **=0.10 ***=0.39
	Women	8	1.98 ± 0.70	2.96 ± 1.71	2.56 ± 0.75	
	Total	30	7.58 ± 2.66	1.59 ± 0.54	3.20 ± 0.99	
Healthy control group	Men	20	1.36 ± 0.46	1.06 ± 0.19	1.14 ± 0.41	*=0.02 **=0.61 ***=0.49
	Women	10	0.36 ± 0.16	0.92 ± 0.22	0.73 ± 0.18	
	Total	30	1.01 ± 0.31	1.00 ± 0.14	1.00 ± 0.27	

The *, ** and *** represent the difference of the fold change expression of the SEMA3A, SEMA4A and SEMA4D between men and women in a specified group.

† The amounts of the semaphprin expression were assessed as fold change and expressed as mean ± SEM.

## Discussion

 The results of the present study showed that the expression of SEMA3A and SEMA4D in patients with CLL was higher than healthy subjects. These results indicate an association between CLL and elevated levels of the aforementioned semaphorins. The molecular mechanisms which are responsible for elevated expression of mentioned semaphorins in CLL patients need to be clarified in more investigations. The SEMA3A expression was also higher in patients with stage I and stage II than in healthy subjects. Moreover, the SEMA3A expression in patients with stage II was higher compared to patients with stage I. These results indicate that the SEMA3A may contribute to tumor progression. 

It has been reported that SEMA3A acts as an anti-tumor agent since it suppresses angiogenesis by competing with VEGF for identical receptors on endothelial cells^6^. In several experimental animal models, it has been indicated that the systemic and intra-tumoral delivery of SEMA3A prevents tumor angiogenesis and tumor development^22^. A negative association was also indicated between SAMA3A expression and development of the non–small cell lung carcinoma and melanoma^23^^,^^24^. On the other hand, the immunosuppressive properties of SEMA3A may account for situations where high amounts of SEMA3A are associated with poor prognosis in urothelial cancer^25^, glioblastoma multiforme^26^, and hepatocellular carcinoma (HCC)^27^. SEMA3A also acts as a chemoattractant for macrophages and promotes tumor-associated macrophage (TAM) infiltration^6^. In hepatocellular carcinoma, a significant correlation was reported between the expression of SEMA3A and the number of intra-tumoral macrophages, especially M2 type of TAM^27^. In agreement with our findings, a positive association was reported between the urinary levels of SAMA3A and tumor stages in patients with urothelial cancer^25^. It has been also indicated that SEMA3A promotes HCC progression through increasing the expression of the gelsolin-like capping protein, galectin-3, enolase 2 and epithelial cell adhesion molecule^28^. In addition, SEMA3A acts as a key factor for the establishment of the cancer stem-like cells originated from Lewis lung carcinoma^29^. The inhibitory effects of anti-SEMA3A antibody on the glioblastoma growth was also indicated^30^. Therefore, it is worthy to investigate the therapeutic potentials of anti-SEMA3A antibody for treatment of cancers. However, as previously mentioned, there are opposite roles played by SEMA3A in cancers. It is important to note that the roles of SEMA3A are dependent on its origination. Although endogenous SEMA3A produced in tumor microenvironment may act as a cancer enhancer, exogenous SEMA3A may act as a tumor inhibitor agent^6^.

The results presented here indicate that the expression of SEMA4D in both subgroups of CLL patients with stage I and stage II was significantly higher than healthy subjects. However, the expression of SEMA4D did not significantly differ between patients who were in stage I and those who were in stage II. These data indicate that the SEMA4D may have differential roles in CLL establishment and progression. SEMA4D is largely expressed by a number of solid tumors such as prostate, ovarian, lung, glioma and sarcoma^31^. The expression of SEMA4D is mainly related with tumor aggressiveness and poor outcome^31^^,^^32^. In pancreatic cancer, SEMA4D produced by tumor-infiltrating lymphocytes enhances tumor cell movement^33^. Moreover, macrophages were reported as the major sources of SEMA4D in breast cancer^34^^,^^35^. The SEMA4D role in tumor-induced angiogenesis was demonstrated particularly for oral squamous cell carcinomas (OSCC). The tumors expressing high amounts of SEMA4D are more aggressive, more difficult to treatment, and have a poor prognosis^32^^,^^36^. SEMA4D may contribute to the immunosuppression through induction of the Treg cells, myeloid-derived suppressor cells and M2 macrophages^31^. The SEMA4D-mediated immunosuppression may play an essential role in the tumor development. SEMA4D may also be considered as a suitable target for treatment of cancers. 

There was no significant difference between CLL patients and healthy subjects concerning the SEMA4A expression. No significant difference was also observed between patients with stage I and stage II regarding the expression of SEMA4A. The results indicated that SEMA4A might possibly have no association with CLL neither with its establishment nor with its progression. 

In normal physiological conditions, SEMA4A is produced by endothelial cells and Plexin-D1 acts as a functional receptor of this semaphoring on these cells. SEMA4A–Plexin-D1 axis has inhibitory effects on the VEGF-induced angiogenesis^12^. Various immunomodulatory effects were also attributed to SEMA4A. During the DCs-T cell communication, SEMA4A on DCs directly binds to TIM-2 on T cells and enhances the activation of antigen-specific T cells^37^. SEMA4A increases the Th1 cell differentiation through a TIM-2 dependent manner. In SEMA4A-deficient mice, Th1 cell-related responses are impaired, while Th2 cell-mediated responses are increased^12^. In addition, SEMA4A-deficient CD8^+^ T cells exhibit defect in cytokine production and expression of effector molecules such as Granzyme B, Perforin, and FasL^38^. SEMA4A also directly reacts with the receptor neuropilin-1 on Treg cells and enhances their function and survival^12^^,^^39^. Furthermore, SEMA4A induces macrophage movement in a dose-dependent process^12^. According to the mentioned immunomodulatory effects attributed to SEMA4A, it seems that the anti-tumoral properties of this semaphorin may be greater than its pro-tumorigenic influences. 

The results of the present study also indicated that the expression of SEMA3A was higher in men than in women either in CLL patients or controls. There is no study concerning the influence of gender on the SEMA3A expression. In one study conducted in patients with glial tumors, it was observed that the expression of SEMA3C and SEMA3F were higher in men than women^40^. These findings suggest the possible influence of gender-related hormones on the expression of some members of the semaphorin family such as SEMA3A. The expression of SEMA3A and SEMA4D in men and women with CLL was significantly higher than control individuals of the same gender. These data suggest that SEMA3A and SEMA4D may contribute to CLL development in both genders.

Determination of semaphorin expression at the protein levels, assessment of other semaphorins, and evaluation of the clinical values of semaphorins such as their association with CLL prognosis are interesting topics for future investigations. 

## CONCLUSION

 In conclusion, the results of this study indicated elevated amounts of SEMA3A and SEMA4D expression in patients with CLL. The expression of SEMA3A was influenced by tumor stage and gender of participants. The clinical values of semaphorins in CLL need to be considered in future investigations. 
